# ﻿Two new species of the *Drawida japonica* species complex (Oligochaeta, Moniligastridae) from East Asia delimited by integrative taxonomic methods

**DOI:** 10.3897/zookeys.1264.170881

**Published:** 2025-12-19

**Authors:** Min Liu, Pu Miao, Zheng Liu, Nonillon M. Aspe, Yufeng Zhang, Huifeng Zhao

**Affiliations:** 1 Hebei Key Laboratory of Animal Diversity, College of Life Science, Langfang Normal University, Langfang 065000, China Langfang Normal University Langfang China; 2 College of Life Science, Shenyang Normal University, Shenyang 110034, Liaoning, China Shenyang Normal University Shenyang China; 3 Henan Province Tobacco Company, Luoyang 471000, China Henan Province Tobacco Company Luoyang China; 4 College of Environment and Life Sciences, Mindanao State University at Naawan, Naawan 9023, Misamis Oriental, Philippines Mindanao State University at Naawan Naawan Philippines

**Keywords:** Earthworm, molecular delimitation, Moniligastridae, phylogeny, taxonomy

## Abstract

The *Drawida japonica* (Michaelsen, 1892) species complex is a cosmopolitan earthworm group that is widely distributed throughout Asia, and has a high degree of diversity. Nonetheless, the species composition of this species complex remains ambiguous due to limited taxonomic investigation. An integrative taxonomic approach, incorporating both morphological and molecular datasets, is herein applied to elucidate the *D. japonica* species complex across East Asia, with the objective of delimiting putative new species. External and internal morphological characters were examined for taxonomic identification. For molecular phylogenetic analysis, one mitochondrial marker, the cytochrome c oxidase subunit I (COI), and three nuclear loci, namely 28S rRNA (28S), A-kinase anchor protein 17A (AKAP17), and flavin adenine dinucleotide synthetase 1 (FLAD1) were used. Species delimitation was performed using three complementary methodological frameworks: Assemble Species by Automatic Partitioning (ASAP), the Generalized Mixed Yule Coalescent (GMYC) phylogenetic approach, and Bayesian Phylogenetics and Phylogeography (BPP). Congruent species boundaries were recovered across all analyses, with the single exception of the GMYC model applied to the mitochondrial COI data set. Furthermore, the interspecific K2P genetic distance exceeded 15%. This study has delimited two new species, namely *D. henanensis***sp. nov.** and *D. sinensis***sp. nov.** The two new species represent the first additions to the species complex in the past decade, thereby significantly contributing to our understanding of *Drawida* earthworm diversity in Asia.

## ﻿Introduction

Earthworms play a pivotal role in maintaining soil health. They facilitate aeration, increase porosity, expedite nutrient cycling, and preserve the functionality of soil ecosystems, thereby contributing to environmental integrity (Scullion and Malik 2000). However, earthworm taxonomy is constrained by limited morphological diagnostic characters and the widespread occurrence of pronounced morphological conservatism among congeneric or even confamilial taxa—an issue exemplified within the family Moniligastridae Claus, 1880 (Pérez-Losada et al. 2009; Blakemore et al. 2014; Liu et al. 2025).

The scientific investigation of Moniligastridae commenced with the initial description of *Moniligaster deshyaesi* Perrier, 1872. According to the most recent literature, 181 valid species in five genera have been recorded in this family (Narayanan et al. 2024a, 2024b, 2025; Liu et al. 2025). However, the taxonomy of this family is often problematic because of the presence of numerous visually similar species groups (Blakemore and Kupriyanova 2010; Blakemore et al. 2014; Liu et al. 2025). Moreover, certain species within Moniligastridae are characterized by ambiguous boundaries, with numerous species grouped into species complexes such as the *Drawida ghilarovi* Gates, 1969 species complex, which either regarded as divergent morphological manifestations of a singular *nepalensis* species or possess ambiguous taxonomic status, introducing complexity to the taxonomy of this family (Gates 1972; Blakemore et al. 2014; Liu et al. 2025). Furthermore, inadequate morphological descriptions of moniligastrids in earlier and current literatures coupled with persistent synonymy issues, have further exacerbated the challenges of species identification (Blakemore and Kupriyanova 2010; Blakemore et al. 2014; Narayanan et al. 2024a). Thus, the incorporation of molecular data, such as the mitochondrial genome (mitogenome) and nuclear marker 28S rRNA (28S), can facilitate the identification of morphologically similar species, as well as the reconstruction of the phylogenetic relationships of target taxa (Blakemore and Kupriyanova 2010; Bernt et al. 2013; Osigus et al. 2013; Narayanan et al. 2024b; Liu et al. 2025). Moreover, it can facilitate the investigation of the processes underlying earthworm species diversification and dispersion (Marchán et al. 2022).

The genus *Drawida* Michaelsen, 1900 is characterized by its notable species richness and wide distribution within the Moniligastridae (Gates 1972; Jamieson 1977). To date, *Drawida* comprises 151 valid species (Narayanan et al. 2024a, 2024b; Liu et al. 2025), with its natural distribution encompassing regions extending from southern to southeastern and eastern Asia (Gates 1936, 1972; Easton 1981; Blakemore 2012; Anderson et al. 2017; Narayanan et al. 2017, 2023; Zhang et al. 2020). Among these species, *D. japonica* sensu lato (Michaelsen, 1892) is one of the most widely distributed species, with a broad range that encompasses nearly the entire distribution of *Drawida* (Misirlioğlu et al. 2023; Narayanan et al. 2024a). Blakemore et al. (2014) posited that *D. japonica* falls within the scope of a species complex, akin to the *D. nepalensis* Michaelsen, 1907 species complex and *D. ghilarovi* species complex (Liu et al. 2025). This assertion is supported by substantial evidence from both morphological and molecular data, which collectively suggests the potential existence of numerous unidentified species or subspecies within *D. japonica* (Blakemore et al. 2014). In accordance with the observation of similar distribution range and comparable morphological characteristics, the *D. japonica* species complex may encompass the known species that belong to this group, which include *D. japonica* sensu stricto (s. s.), *D. calebi* Gates, 1945; *D. koreana* Kobayashi, 1938; *D. siemsseni* Michaelsen, 1910; *D. jeholensis* Kobayashi, 1940; *D. moriokaensis* Ohfuchi, 1938, *D. keikiensis* Kobayashi, 1938 and *D. companio* Blakemore, 2014 (Stephenson 1923; Kobayashi 1940a; Blakemore et al. 2014; Narayanan et al. 2024a). *Drawida japonica* s. s. share certain similarities with these closely related regional congeners, such as *D. koreana*, *D. siemsseni*, *D. companio*, *D. moriokaensis* and *D. keikiensis* with clitellum in X–XIII; *D. koreana*, *D. jeholensis* and *D. moriokaensis* with two or three gizzards.

The precise taxonomic identification and geographical distribution of *D. japonica* species complex remain ambiguous. Gates (1972) initially proposed that *D. japonica* originated from the Indian Himalayas, as well as regions of Southwest China. Subsequently, Easton (1981) documented the occurrence of specimens in Japan and Korea, which were identified within the distribution range of this taxon. The presence of the complex has also been documented in a large area of mainland China (Michaelsen 1927; Chen 1933, 1936, 1946; Gates 1935, 1939; Kobayashi 1940b; Zhang et al. 2012, 2016; Bi et al. 2025), and Blakemore (2012) proposed that it may have originated from Taiwan and subsequently spread to Japan. Additionally, the minimal morphological variations exhibited in the *D. japonica* species complex render taxonomic analyses based exclusively on morphological evidence as a highly taxing task (Blakemore et al. 2014). This underscores the necessity of incorporating additional taxonomic methodologies, including molecular or ecological data, to facilitate the discovery of new species. However, the scope of molecular study on *D. japonica* has been limited in previous studies. Notably, only studies from India, Japan, South Korea, and China have provided a modest amount of molecular data (Huang et al. 2007; Blakemore and Kupriyanova 2010; Blakemore et al. 2014), of which are primarily restricted to the mitochondrial marker cytochrome c oxidase subunit I (COI). On the other hand, 16S rRNA (16S) data for India was provided by Kumari et al. (2021).

Mitochondrial DNA has been extensively utilized in taxonomic studies, especially COI (Thakur et al. 2021; Marchán et al. 2023; Seesamut et al. 2024; Aspe et al. 2025). However, it is inherited as a maternal evolutionary and rapid genetic marker, providing only a single estimate of the species tree. Consequently, it may not accurately reflect the evolutionary history of species (Hurst and Jiggins 2005; White et al. 2008; Ballard and Whitlock 2022; Chen et al. 2023). In contrast, nuclear markers are universal and composed of highly conserved and variable regions that reflect parental genetic backgrounds, and evolve comparatively more slowly than mitochondrial markers (Patwardhan et al. 2014). This attribute renders them more precise than mitochondrial markers in determining the trajectory of species diversification, and their application in numerous molecular species delimitation studies of earthworms has been well documented in the literatures (James and Davidson 2012; Aspe and James 2018; Plytycz et al. 2018; Liu et al. 2025).

This study employs an integrative taxonomy approach, analyzing the specimens of the *D. japonica* species complex collected from Central China using morphological and molecular data. In addition to the use of the mitochondrial COI gene fragment, three nuclear gene fragments [28S, A-kinase anchor protein 17A (AKAP17), and flavin adenine dinucleotide synthetase 1 (FLAD1)] were also utilized. The application of molecular species delimitation methods, including the Assemble Species by Automatic Partitioning (ASAP), Generalized Mixed Yule Coalescent (GMYC), and the Bayesian Phylogenetics and Phylogeography (BPP), in conjunction with the phylogenetic method, has enabled the delimitation of putative species in the *D. japonica* species complex.

## ﻿Materials and methods

### ﻿Sampling

The collection of earthworm samples was conducted in May 2023 in Luoning County, Luoyang Prefecture, Henan Province, China (34.4364°N, 111.6368°E). The samples were obtained by digging and hand-sorting. Following collection, the samples were preserved in 100% ethanol for subsequent morphological and molecular analyses. A total of 45 samples were deposited in the Hebei Key Laboratory of Animal Diversity, Langfang Normal University, Langfang, China (**C-HLU**).

### ﻿Morphological examination and taxonomic identification

A total of 14 clitellate samples were examined using a stereomicroscope (ZEISS) and ZEN 3.3.pro software to capture images and examine the detail of both external and internal characteristics. The examination of adult earthworms was chiefly based on body length, width, and color; prostomium type; arrangement of setae; position and morphology of the clitellum; male pores; spermathecal pores; genital markings; and internal organs, including testis sacs, spermathecae, ovisacs, prostates, and gizzards. The generic diagnoses and taxonomic assignments follow Blakemore et al. (2014), Michaelsen (1892) and Xu and Xiao (2011). All measures are based on the materials that preserved in alcohol. The comparison of key morphological characteristics of the *D. japonica* species complex is shown in Table 1.

**Table 1.** Key morphological characteristics of the *Drawida japonica* species complex (NA: not available).

Characteristic	* D. japonica* s. s.	* D. henanensis* sp. nov.	* D. sinensis* sp. nov.	* D. koreana*	* D. siemsseni*	* D. calebi*	* D. jeholensis*	* D. companio*	* D. moriokaensis*	* D. keikiensis*
**Color**	Grey/Pale	Grey	Grey	Dark blue	buff	Unpigmented	Whitish grey	Dark bluish	Grey	Grey
**Length (mm)**	28–70	24–47	41.8–52	63–100	110	35–83	52–66	80+	65–100	40–54
**Width (mm)**	2–4.5	1.8–3	3.5–4	≤ 4	2–4	2–4.5	≤ 3.5	NA	≤ 3.9	≤ 2.5
**Genital markings**	7–13, unpaired	7–11, unpaired	7–11, unpaired	7–12, irregular	7–12, irregular	7–13, variously	7–11	8	None	None
**Clitellum**	9,10–13,½14	10–13; light grey	10–13; grayish white	10–13; pinkish	10–13	NA	9–14	10–13	10–13	10–13
**Sperm ducts**	Long coiled	Medium coiled	Tight coiled	Short less coiled	Long coiled	Long coiled	Convoluted	Moderately long	Medium coiled	Long coiled
**Sperm atrium**	thumb-like and relatively large in 8	Long sac-like and small in 8	Long sac-like and small in 8	Short, sac-like in 7	Present in 8	Conical in 8	Large in 7/8	Small	Small in 7/8	7/8
**Form of male pores**	Stubby flab on 10 near 10/11	Penis-like overhanging on 10/11	Raised on the 10/11	Penis-like on 10 near10/11	10/11	Short penis at 10/11	10/11	10/11	Short and stout Penis in pouch in 10/11	Penis in pouch in 10/11
**Female pore**	11/12; near b-line	Absent	Absent	12; in b-line	NA	NA	12; in ab-line	12; in b-line	12,near 11/12	11/12; in b-line
**Ovisacs**	11/12–16	12–20	12–15	12–18, seldom 22 or 23	NA	Extend to 20	Extend to 16–20	Extend to 14	Ovarian chamber in 10/11/12 with egg sacs	10/11/12–16 or 22
**Gizzard segments**	2 or 3; (11)12–13,14	2 or 3; (11)12–13,14 (16)	2; 11–13	2 or 3; 12–14	6	2–4; 12–17	2 or 3; 12–13 or 11–13	3; 12–14	2–3; 10, 11–12?	3–4; 12,13–15
**Vas deferens**	Coiled and twisted	Slender and less coiled	Long coiled	Loosely twisted	NA	Short coiled	Short	Coiled	Coiled	Short coiled
**Prostate**	Club-shaped and erect	Elliptical white and thick disc-shaped	Long white and thick	Thumb-shaped	NA	Sessile and smooth	Small and rugose	Small and white	NA	Small and short, but broad and warty on surface

### ﻿DNA extraction, amplification, and sequencing

The total genomic DNA of the samples was extracted from the posterior part using the TIANamp Genomic DNA Kit (TIANGEN, Beijing, China) following the manufacturer’s instructions. The quantification of DNA concentrations was conducted using a BIO DL MicroDrop spectrophotometer (Beijing, China).

The COI and 28S were amplified using polymerase chain reaction (PCR). Another two nuclear genes, AKAP17 and FLAD1, were amplified using nested PCR. The mixture (total volume of 25 μl) contained 1 μl of DNA and 24 μl of PCR mix, which consisted of 17.25 μl of sterile ddH_2_O, 2.5 μl of Easy Taq Buffer, 0.25 μl of Easy Taq Polymerase (TransGen Biotech Co., Ltd, Beijing, China), 1 μl of each forward and reverse primer (10 μM), and 2.0 μl of dNTPs. The primers utilized are listed in Table 2. Notably, in the context of nested PCR, the second-round reactions utilized 1 μl of PCR products derived from the first round as DNA templates. The PCR protocol for COI includes an initial denaturation step at 95 °C for 5 min, followed by 35 cycles at 95 °C for 30 sec, an annealing step at 51 °C for 30 sec, an extension step at 72 °C for 45 sec, and a final extension step at 72 °C for 5 min. The annealing temperature of 28S was 54 °C, and the annealing temperature was 48 °C for both AKAP17 and FLAD1. The PCR products were examined by electrophoresis in a 1% agarose gel and sent to Tianyi Huiyuan Biotechnology Co., Ltd. (Beijing, China) for sequencing. The sequences were aligned and edited using MEGA 5 (Tamura et al. 2011). All sequences and annotations were submitted to GenBank and the accession numbers are shown in Table 3.

**Table 2.** Primers used for PCR and sequencing.

Marker	Primer	Sequence (5’-3’)	Round	Source
COI	LCO1490	GGTCAACAAATCATAAAGATATTGG		Folmer et al. (1994)
	HCO2198	TAAACTTCAGGGTGACCAAAAAATCA		
	COIE	TATACTTCTGGGTGTCCGAAGAATCA		Bely and Wray (2004)
28S	28sF1	GAGTACGTG AAACCGTCTAG		Pérez-Losada et al. (2009)
	28sR1	CGTTTCGTCCCCAAGGCCTC		
AKAP17	AKAP17-F1	AAYTGGGARGTNATGGARAA	Round 1	Liu et al. (2025)
	AKAP17-R1	TCYTTRAACATNARYTTCAT		
	AKAP17-F2	AARATGATHAARCCNGAYCARTT	Round 2	
	AKAP17-R2	GCYTTNACRAANCCCATRTAYTC		
FLAD1	FLAD1-F1	GGNCCNACNCAYGAYGAYAT	Round 1	
	FLAD1-R1	TTNGGRTGNGTRTTYTCCAT		
	FLAD1-F2	TGYAARGCNTTYTTYGGNACNGA	Round 2	
	FLAD1-R2	TTNACNCKCATRAAYTCNGGCCA		

**Table 3.** Information on *Drawida* species included in this analysis.

Specimen ID	Species	Location	GPS coordinates	Accession Number			
				COI	28S	AKAP17	FLAD1
HNLN-GR-I1_02	* D. henanensis* sp. nov.	China: Henan: Luoyang: Luoning	34.4364°N, 111.6368°E	PQ288546	PQ432438	PQ452820	–
HNLN-GR-I2_23	* D. henanensis* sp. nov.	China: Henan: Luoyang: Luoning	34.4364°N, 111.6368°E	PQ288549	PQ432440	PQ452819	PQ452822
HNLN-GR-I2_19	* D. henanensis* sp. nov.	China: Henan: Luoyang: Luoning	34.4364°N, 111.6368°E	PQ288547	–	–	–
HNLN-GR-I2_20	* D. henanensis* sp. nov.	China: Henan: Luoyang: Luoning	34.4364°N, 111.6368°E	PQ288548	–	–	–
HNLN-GR-I2_24	* D. henanensis* sp. nov.	China: Henan: Luoyang: Luoning	34.4364°N, 111.6368°E	PQ288550	–	–	–
HNLN-GR-I2_25	* D. henanensis* sp. nov.	China: Henan: Luoyang: Luoning	34.4364°N, 111.6368°E	PQ288551	–	–	–
HNLN-GR-I2_26	* D. henanensis* sp. nov.	China: Henan: Luoyang: Luoning	34.4364°N, 111.6368°E	PQ288552	PQ432439	PQ452818	PQ452823
HNLN-GR-I2_27	* D. henanensis* sp. nov.	China: Henan: Luoyang: Luoning	34.4364°N, 111.6368°E	PQ288553	PQ432436	PQ452817	PQ452824
HNLN-GR-I2_28	* D. henanensis* sp. nov.	China: Henan: Luoyang: Luoning	34.4364°N, 111.6368°E	PQ288554	–	–	–
HNLN-GR-I2_29	* D. henanensis* sp. nov.	China: Henan: Luoyang: Luoning	34.4364°N, 111.6368°E	PQ288555	PQ432437	PQ452816	PQ452825
HNLN-GR-I2_30	* D. henanensis* sp. nov.	China: Henan: Luoyang: Luoning	34.4364°N, 111.6368°E	PQ288556	–	–	–
HNLN-GR-I2_31	* D. henanensis* sp. nov.	China: Henan: Luoyang: Luoning	34.4364°N, 111.6368°E	PQ288557	–	–	–
HNLN-GR-I2_32	* D. henanensis* sp. nov.	China: Henan: Luoyang: Luoning	34.4364°N, 111.6368°E	PQ288558	–	–	–
HNLN-GR-I2_44	* D. henanensis* sp. nov.	China: Henan: Luoyang: Luoning	34.4364°N, 111.6368°E	PQ288562	–	–	–
HNLN-GR-I2_45	* D. henanensis* sp. nov.	China: Henan: Luoyang: Luoning	34.4364°N, 111.6368°E	PQ288563	–	–	–
HNLN-GR-I2_46	* D. henanensis* sp. nov.	China: Henan: Luoyang: Luoning	34.4364°N, 111.6368°E	PQ288564	–	–	–
HNLN-GR-I2_47	* D. henanensis* sp. nov.	China: Henan: Luoyang: Luoning	34.4364°N, 111.6368°E	PQ288565	–	–	–
HNLN-GR-I2_48	* D. henanensis* sp. nov.	China: Henan: Luoyang: Luoning	34.4364°N, 111.6368°E	PQ288566	–	–	–
HNLN-GR-I2_49	* D. henanensis* sp. nov.	China: Henan: Luoyang: Luoning	34.4364°N, 111.6368°E	PQ288567	–	–	–
HNLN-GR-I2_50	* D. henanensis* sp. nov.	China: Henan: Luoyang: Luoning	34.4364°N, 111.6368°E	PQ288568	–	–	–
HNLN-GR-I2_51	* D. henanensis* sp. nov.	China: Henan: Luoyang: Luoning	34.4364°N, 111.6368°E	PQ288569	PQ432444	–	–
HNLN-GR-I2_52	* D. henanensis* sp. nov.	China: Henan: Luoyang: Luoning	34.4364°N, 111.6368°E	PQ288570	–	–	–
HNLN-GR-I2_53	* D. henanensis* sp. nov.	China: Henan: Luoyang: Luoning	34.4364°N, 111.6368°E	PQ288571	PQ432445	PQ452812	–
HNLN-GR-I2_54	* D. henanensis* sp. nov.	China: Henan: Luoyang: Luoning	34.4364°N, 111.6368°E	PQ288572	PQ432446	PQ452811	PQ452821
HNLN-GR-I2_55	* D. henanensis* sp. nov.	China: Henan: Luoyang: Luoning	34.4364°N, 111.6368°E	PQ288573	–	–	–
HNLN-GR-I2_56	* D. henanensis* sp. nov.	China: Henan: Luoyang: Luoning	34.4364°N, 111.6368°E	PQ288574	–	–	–
HNLN-GR-I2_57	* D. henanensis* sp. nov.	China: Henan: Luoyang: Luoning	34.4364°N, 111.6368°E	PQ288575	–	–	–
HNLN-GR-I2_58	* D. henanensis* sp. nov.	China: Henan: Luoyang: Luoning	34.4364°N, 111.6368°E	PQ288576	–	–	–
HNLN-GR-I3_03	* D. henanensis* sp. nov.	China: Henan: Luoyang: Luoning	34.4364°N, 111.6368°E	PQ288579	–	–	–
HNLN-GR-I3_06	* D. henanensis* sp. nov.	China: Henan: Luoyang: Luoning	34.4364°N, 111.6368°E	PQ288580	–	–	–
HNLN-GR-I3_29	* D. henanensis* sp. nov.	China: Henan: Luoyang: Luoning	34.4364°N, 111.6368°E	PQ288581	PQ432449	–	–
HNLN-GR-I3_43	* D. henanensis* sp. nov.	China: Henan: Luoyang: Luoning	34.4364°N, 111.6368°E	PQ288582	–	–	–
HNLN-GR-I3_46	* D. henanensis* sp. nov.	China: Henan: Luoyang: Luoning	34.4364°N, 111.6368°E	PQ288583	–	–	–
HNLN-GR-I4_14	* D. henanensis* sp. nov.	China: Henan: Luoyang: Luoning	34.4364°N, 111.6368°E	PQ288585	–	–	–
HNLN-GR-I4_17	* D. henanensis* sp. nov.	China: Henan: Luoyang: Luoning	34.4364°N, 111.6368°E	PQ288588	–	–	–
HNLN-GR-I4_18	* D. henanensis* sp. nov.	China: Henan: Luoyang: Luoning	34.4364°N, 111.6368°E	PQ288589	–	–	–
EF077597	* D. henanensis* sp. nov.	China, Huang et al. 2007	–	EF077597	–	–	–
EF077598	* D. henanensis* sp. nov.	China, Huang et al. 2007	–	EF077598	–	–	–
EF077599	* D. henanensis* sp. nov.	China, Huang et al. 2007	–	EF077599	–	–	–
EF077600	* D. henanensis* sp. nov.	China, Huang et al. 2007	–	EF077600	–	–	–
w13	* D. sinensis* sp. nov.	South Korea, Blakemore et al. 2014	–	w13	–	–	–
HNLN-GR-I2_40	* D. sinensis* sp. nov.	China: Henan: Luoyang: Luoning	34.4364°N, 111.6368°E	PQ288561	PQ432443	PQ452813	PQ452830
HNLN-GR-I2_34	* D. sinensis* sp. nov.	China: Henan: Luoyang: Luoning	34.4364°N, 111.6368°E	PQ288559	PQ432441	PQ452815	PQ452828
HNLN-GR-I3_47	* D. sinensis* sp. nov.	China: Henan: Luoyang: Luoning	34.4364°N, 111.6368°E	PQ288584	–	–	–
HNLN-GR-I4_20	* D. japonica* s. s.	China: Henan: Luoyang: Luoning	34.4364°N, 111.6368°E	PQ288590	–	–	–
HNLN-GR-I4_15	* D. japonica* s. s.	China: Henan: Luoyang: Luoning	34.4364°N, 111.6368°E	PQ288586	–	–	–
HNLN-GR-I4_16	* D. japonica* s. s.	China: Henan: Luoyang: Luoning	34.4364°N, 111.6368°E	PQ288587	–	–	–
HNLN-GR-I3_02	* D. japonica* s. s.	China: Henan: Luoyang: Luoning	34.4364°N, 111.6368°E	PQ288578	PQ432448	PQ452809	PQ452827
HNLN-GR-I2_36	* D. japonica* s. s.	China: Henan: Luoyang: Luoning	34.4364°N, 111.6368°E	PQ288560	PQ432442	PQ452814	PQ452829
HNLN-GR-I3_01	* D. japonica* s. s.	China: Henan: Luoyang: Luoning	34.4364°N, 111.6368°E	PQ288577	PQ432447	PQ452810	PQ452826
GQ500902	* D. japonica* s. s.	Japan: Shiga-ken: Hikone-shi	35.16°N, 136.16°E	GQ500902	–	–	–
JET101_11	* D. japonica* s. s.	Japan, Blakemore et al. 2014	–	JET101_11	–	–	–
JET116_11	* D. japonica* s. s.	Japan, Blakemore et al. 2014	–	JET116_11	–	–	–
JET117_11	* D. japonica* s. s.	Japan, Blakemore et al. 2014	–	JET117_11	–	–	–
KF205976	* D. japonica* s. s.	China: Shanghai	31.1477°N, 121.3613°E	KF205976	–	–	–
LFSF_003	* D. gisti*	China: Hebei: Langfang	39.5222°N, 116.6644°E	PQ675805	PQ675807	PQ683865	PQ683867
E07_01	* D. gisti*	China: Tianjin: Binhai	39.0800°N, 117.6963°E	PQ675804	PQ675808	PQ683864	PQ683866

### ﻿Molecular species delimitation analyses

Sequences of closely related taxa included in the molecular analyses were obtained from GenBank (Table 3). Genetic distances were calculated using the Kimura-2-Parameter (K2P) model (Kimura 1980) (Table 4). A comparison of molecular species delimitation with the results of the morphological identification was performed using Assemble Species by ASAP (Puillandre et al. 2021), GMYC (Fujisawa and Barraclough 2013), and BPP (Yang and Rannala 2010). The results of these methods can be cross-verified to improve the accuracy of species delimitation (Gao et al. 2021).

**Table 4.** The genetic distance of K2P of the species of *Drawida* (values in %, indicating intra-species genetic distance in parentheses).

	Species	1	2	3	4	5	6	7	8	9
1	* D. henanensis* sp. nov.	(0–6.5)								
2	* D. japonica* s. s.	15.4–18.0	(0–9.6)							
3	* D. sinensis* sp. nov.	15.4–18.8	15.1–17.1	(0.5–9.8)						
4	* D. gisti*	19.3–22.1	18.5–20.0	17.0–17.9	(0.2)					
5	* D. hattamimizu*	19.3–22.3	18.1–20.5	18.1–20.2	19.1–19.7	(0.2)				
6	* D. ghilarovi*	18.1–21.4	18.7–20.5	16.4–19.3	17.6–18.7	14.5–16.5	(2.1)			
7	* D. ganini*	18.7–20.2	21.7–22.7	20.2–23.6	22.7–23.7	18.5–19.7	14.0–15.1	(0.9)		
8	* D. koreana*	17.1–18.4	7.4–8.3	14.5–15.7	18.3	19.6–19.9	18.5–19.1	20.2–20.8	(0.5)	
9	* D. barwelli*	21.8–25.0	22.3–23.6	18.5–20.6	20.4	20.6–20.9	19.6–20.0	20.6–0.9	22.6–22.9	(0)

The ASAP and GMYC methods were applied to COI and 28S separately to preliminarily infer species hypotheses. ASAP is an automatic program based on DNA barcode gaps that uses the principle that the intraspecific variation is usually smaller than the interspecific variation. It defines species by observing the divergence between DNA sequences. ASAP was performed at the online portal (https://bioinfo.mnhn.fr/abi/public/asap).

The GMYC analysis was executed using split v. 1.0-19 package (Ezard et al. 2017) in R, with the objective of delineating independently evolving species through the utilization of single-locus data. The BEAST analysis was conducted using the BEAST v. 1.7.5 package (Drummond et al. 2012), and the input file was prepared in BEAUTi, incorporating the Yule Process tree prior and a lognormal relaxed clock model. Subsequently, newick-formatted ultrametric, bifurcated, and rooted trees were generated by TreeAnnotator (included in the BEAST package) as inputs for the GMYC analysis. The final tree was visualized in FigTree v. 1.4.4 (Rambaut 2022).

The BPP method was executed based on the four loci (COI, 28S, AKAP17 and FLAD1) to verify the putative species. This approach is well suited for analyzing multi-locus sequence data under the multispecies coalescent model (MSC), which employs trans-model Markov chain Monte Carlo (MCMC) to calculate posterior probabilities of various species trees (Yang 2002, 2015; Rannala and Yang 2003; Flouri et al. 2018). Two analyses were conducted in BPP v. 4.7. The first analysis, designated as A00, involved the estimation of parameters under the MSC on a fixed species phylogeny. This analysis was used to generate the posterior distribution of the parameters, which included the population size parameters (*θs*) and the species divergence times (*τs*) (Yang 2002; Rannala and Yang 2003; Burgess and Yang 2008). The second analysis, designated as A10, involved the species delimitation using a fixed guide tree. The within-model parameter posterior was generated by running A00 (Yang and Rannala 2010; Rannala and Yang 2013). The measurement of both the *θs* and *τ_s_* parameters was achieved through the utilization of the sequence distance or the expected number of mutations or substitutions per site (Flouri et al. 2018). The inverse-gamma prior IG (3, 0.005) was assigned for *θs* and the divergence time at the root of the species tree (*τ_0_*), with a mean of 0.005/(3 - 1) = 0.0025. Moreover, priors for the remaining *τ* parameters estimated for internal nodes were specified by a uniform Dirichlet distribution (Yang and Rannala 2010).

### ﻿Phylogenetic analysis

The maximum likelihood (ML) and Bayesian inference (BI) methods were employed to construct phylogenetic trees, with analyses based on mitochondrial (COI), and the mito-nuclear combined dataset (COI, 28S, AKAP17 and FLAD1). *Drawida gisti* was designated as the outgroup. The determination of the most suitable substitution model for each gene employed in phylogenetic analysis was performed using jModelTest 2.1 (Darriba et al. 2012) based on the Akaike Information Criterion: TPM3uf+I+G for COI, and GTR+I+G for the combined dataset. ML analysis was executed using the RAxML 8.0 software (Stamatakis 2014), with 1,000 bootstrapping replicates and default parameter settings. The optimal tree was constructed through the use of the RAxML program, with the GTRGAMMA model. BI analysis was performed using MrBayes v. 3.2.6 (Ronquist et al. 2012), and run for two million generations, with a sampling frequency of 1,000 generations, to ensure that the average standard deviation of split frequencies was less than 0.01. The results of the p-files of BI were examined in Tracer v. 1.7.2 (Rambaut et al. 2018), and effective sampling size (ESS) values larger than 200 were accepted. The resulting trees were then subjected to visual analysis and subsequent editing using FigTree v. 1.4.4.

### ﻿Abbreviations

**ag** accessory gland;

**gm** genital markings;

**amp** ampulla;

**vd** vas deferens;

**ts** testis sacs;

**sp** spermathecal pore;

**mp** male pore;

**prg** prostate gland;

**cl** clitellum;

**oc** ovary chamber;

**p** prostomium;

**os** prostomium;

**fp** female pore.

## ﻿Results

### ﻿Taxonomy

**Family Moniligastridae Claus, 1880**

**Genus *Drawida* Michaelsen, 1900**

#### ﻿ Drawida henanensis

Taxon classificationAnimaliaMoniligastridaMoniligastridae

Liu & Zhao sp. nov.

D4AE5933-BE9D-5E14-83CD-E4F1EFBAC198

https://zoobank.org/B2B640AF-E77F-43A1-8C0A-DB1CF6CAD29A

Figs 1, 2

##### Type material.

***Holotype*** • HNLN-GR-I2_23 (COI accession number: PQ288549), one clitellate, Luoning County (34.4364°N, 111.6368°E), Luoyang Prefecture, Henan Province, China, 2023-05-18, coll. Huifeng Zhao. ***Paratypes*** • four clitellates, HNLN-GR-I2_27 (COI accession number: PQ288553), HNLN-GR-I2_50 (COI accession number: PQ288568), HNLN-GR-I1_02 (COI accession number: PQ288546) and HNLN-GR-I2_55 (COI accession number: PQ288573), with the same data as the holotype. All types of this new species were deposited in C-HLU.

##### Diagnosis.

Grey when preserved in alcohol. Length 24–47 mm, diameter 1.8–3.0 mm, segments up to 130. Clitellum in X–XIII. Dorsal pores absent. Setae small and closely paired. Spermathecal pores in 7/8, male pores in 10/11, female pores more usually inconspicuous. Genital markings present in VIII–XI, unpaired or absent. Spermathecae in VII–VIII. Testis sacs in IX–X, light yellow, with white and thick prostate glands in X. Ovary chamber in XI, with long ovisacs extending to XX. Gizzards two or three, in (XI) XII–XIII or XIV (XVI).

##### Description.

**Figure 1.**
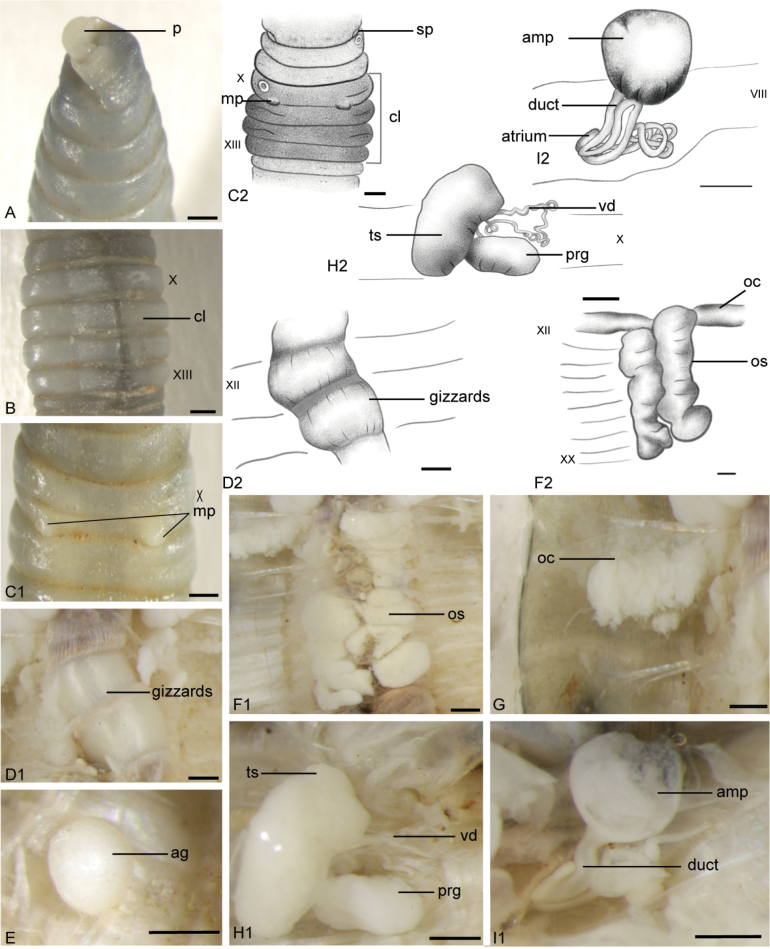
*
Drawida henanensis* sp. nov., holotype (HNLN-GR-I2_23). **A.** Ventral view of the prostomium; **B.** Dorsal view of the clitellum region; **C1, C2.** Ventral view of male pores, genital markings, and spermathecal pores; **D1, D2.** Ventral view of gizzards; **E.** Ventral view of the accessory gland; **F1, F2.** Ventral view of ovisacs; **G.** Ventral view of ovary chamber; **H1, H2.** Left testis sac, with vas deferens and prostate gland; **I1, I2.** Right spermathecae. Scale bars: 0.5 mm.

**Figure 2.**
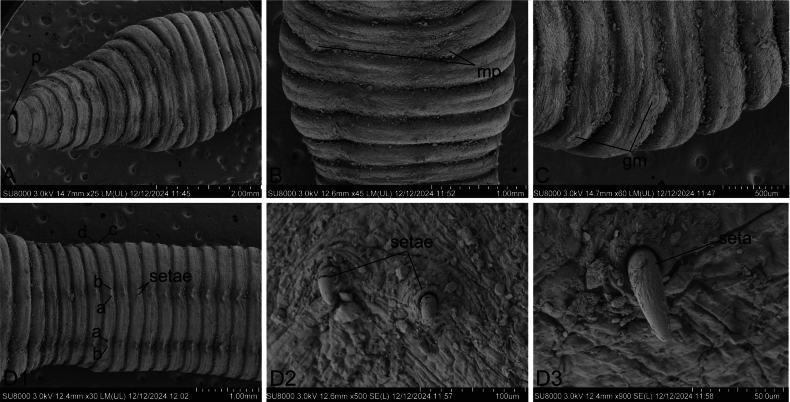
*
Drawida henanensis* sp. nov. (HNLN-GR-I2_27). Electron microscope image showing **A.** Ventral view of the prostomium; **B.** Ventral view of male pores; **C.** Ventral view of genital markings; **D1–D3.** Ventral view of setae.

***External characters***: Size 24–47 mm by 1.8–3 mm, segments ≤ 130, body grey. Prostomium prolobous (Figs 1A, 2A). Clitellum in X–XIII, light grey (Fig. 1B). Dorsal pores absent. Setae small and closely paired (aa = bc) (Fig. 2D1–D3). Spermathecal pores paired in 7/8 in the bc-line, close to c, or usually inconspicuous (Fig. 1C2). Male pores paired in bc-line, close to b, overhanging segment 10/11, penis-like (Figs 1C1, C2, 2B). Female pore absent. Genital markings circular (Fig. 2C), irregularly unpaired in VIII–XI, or absent. Penis absent.

***Internal characters***: Septa 5/6–8/9 thick and muscular, 10/11 and 11/12 surround to form an ovary chamber (Fig. 1G). Ovisacs slightly yellowish, covering gizzards from 11/12 as far back as XX (Fig. 1F1, F2). Gizzards two in XI (XII)–XIII (XIV) or three in XII–XVI (Fig. 1D1, D2). Spermathecae paired in VII–VIII with a spherical-like ampulla, medium coiled duct, and long sac-like and small spermathecal atrium in VIII (Figs 1I1, I2, 8A). Testis sacs light yellow in IX–X, vas deferens slender and less coiled; with elliptical white and thick prostate glands in X (Fig. 1H1, H2). Accessory glands round and stalkless in VII–XI (Fig. 1E), corresponding with genital markings outside.

##### Distribution.

Luoning County (34.4364°N, 111.6368°E), Luoyang Prefecture, Henan Province, China.

##### Habitat.

Around the tobacco field where there is organic fertilizer.

##### Etymology.

The name refers to the possible distribution range in China.

##### Remarks.

The species exhibits significant morphological similarity to *D. japonica* s. s., particularly with respect to body coloration, which is grey when preserved in alcohol, having the male pores pair in 10/11, the testis sacs in IX–X, and the prostate gland in X. However, a notable difference is the clitellum of *D. henanensis* sp. nov. in X–XIII, whereas in (IX) X–XIII or ½XIV in *D. japonica* s. s. The new species exhibits a loose and less coiled vas deferens, as well as an elliptical, white, and thick prostate gland. In contrast, *D. japonica* s. s. is characterized by coiled and twisted vas deferens and a club-shaped and erect prostate gland.

*Drawida henanensis* sp. nov. is also somewhat similar to *D. koreana*, *D. keikiensis*, *D. companio*, *D. moriokaensis*, and *D. siemsseni* in terms of the location of the clitellum (X–XIII). However, the new species is distinguished from both *D. koreana* and *D. siemsseni* by its gray body coloration and medium coiled spermathecal ducts. In contrast, *D. koreana* has a dark blue body and short, less coiled spermathecal ducts, while *D. siemsseni* has a light yellow body and long coiled spermathecal ducts. *D. henanensis* sp. nov. exhibits unpaired genital markings in VII–XI and lacks female pores, while the genital markings of *D. keikiensis* and *D. moriokaensis* are absent, and female pores for the latter species are present in XII and 11/12, respectively. In addition, the new species exhibits ovisacs extending to XX, while the ovisacs of *D. companio* extend to XIV. *D. henanensis* sp. nov. is distinguished from *D. jeholensis* by its relatively diminutive spermathecal atrium in VIII and the absence of female pores, while *D. jeholensis* is characterized by a comparatively substantial spermathecal atrium in 7/8 and the presence of female pores in XII. Furthermore, the new species exhibits loose and less coiled vas deferens of testis sacs, an elliptical, white, and thick disc-shaped prostate, without penis. The new species also differs from *D. calebi* such that the latter is characterized by a short, coiled vas deferens of testis sacs, a sessile and smooth prostate, and a penis that is short at 10/11.

#### ﻿ Drawida sinensis

Taxon classificationAnimaliaMoniligastridaMoniligastridae

Liu & Zhao sp. nov.

38B9B5FF-A57C-5F01-B612-444D7BC98997

https://zoobank.org/3FC645D6-B8D0-46A6-B637-ABAEA1DF547B

Fig. 3

##### Type material.

***Holotype*** • HNLN-GR-I2_40 (COI accession number: PQ288561), one clitellate, Luoning County (34.4364°N, 111.6368°E), Luoyang Prefecture, Henan Province, China, 2023-05-18, coll. Huifeng Zhao. ***Paratypes*** • Two clitellates, HNLN-GR-I2_34 (COI accession number: PQ288559) and HNLN-GR-I3_47 (COI accession number: PQ288584), have the same data as the holotype. All types of this new species were deposited in C-HLU.

##### Diagnosis.

Body light grey. Length 41.8–52 mm, diameter 3.5–4 mm, segments 144–151. Prostomium prolobous. Clitellum in X–XIII. Spermathecal pores in 7/8, male pores in 10/11, and female pore absent. Setae small and closely paired. Spermathecae in VII–VIII with tightly coiled ducts. Testis sacs in IX–X, with long white and thick prostate glands in X. Ovary chamber in XI, and ovisacs in XII–XV. Gizzards two in XI–XIII. Penis absent.

##### Description.

**Figure 3.**
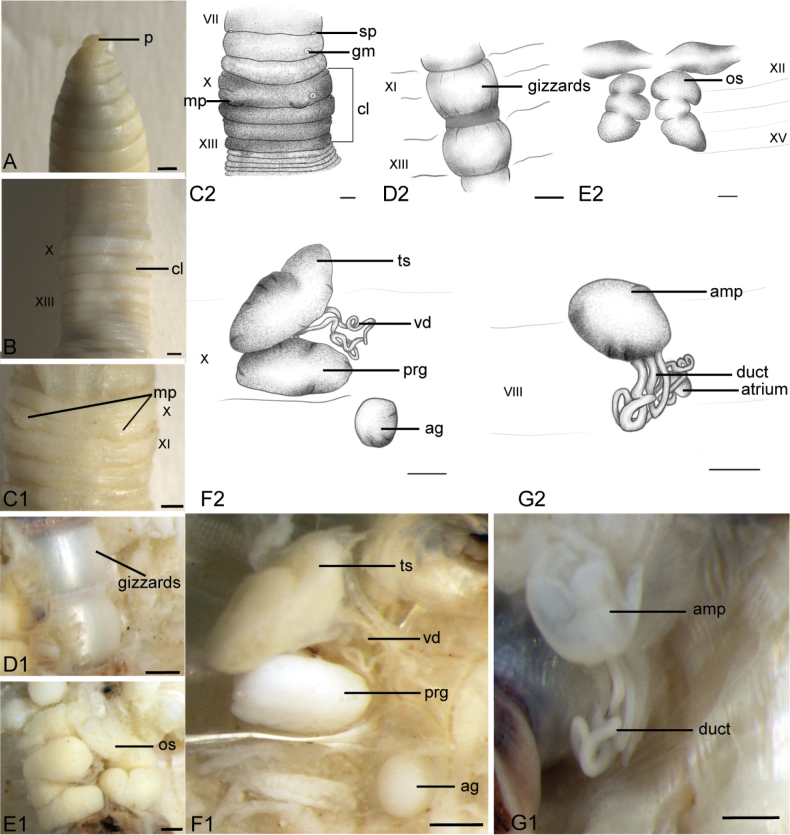
*
Drawida sinensis* sp. nov., holotype (HNLN-GR-I2_40). **A.** Ventral view of the prostomium; **B.** Dorsal view of clitellum region; **C1, C2.** Ventral view male pores, genital markings, and spermathecal pores; **D1, D2.** Ventral view of gizzards; **E1, E2.** Ventral view of ovisacs; **F1, F2.** Left testis sac, vas deferens, prostate gland, accessory gland; **G1, G2.** Right spermathecae. Scale bars: 0.5 mm.

***External characters***: Body light grey when preserved in alcohol. Prostomium prolobous (Fig. 3A). Size 41.8–52 mm by 3.5–4 mm, segments ≤ 151. Clitellum in X–XIII, grayish white (Fig. 3B). Setae small and closely paired (aa = bc). Dorsal pore and female pore absent. Spermathecal pores paired at 7/8, between setae b and c, close b, or inconspicuous (Fig. 3C2). Male pores raised on 10/11 in bc-line close to b, penis absent (Fig. 3C1, C2). Genital markings circular and unpaired in VII–XI.

***Internal characters***: Spermathecae protected by thick and muscular septae (7/8–8/9) in VII–VII, with a circular ampulla and tightly coiled ducts, and long sac-like and small spermathecal atrium in VIII (Figs 3G1, G2, 8B). Testis sacs light yellow at IX–X, with long coiled vas deferens (Fig. 3F1, F2). Long white and thick prostate glands close testis sacs in X (Fig. 3F1, F2). Ovary chamber in XI with yolk-yellow ovisacs extends to XV (Fig. 3E1, E2). Gizzards two in XI–XIII, milky white and covered by ovisacs (Fig. 3D1, D2). Accessory glands are round and stalkless, corresponding with genital markings outside in VII–XI.

##### Distribution.

Henan Province, China, and South Korea (Blakemore et al. 2014).

##### Habitat.

Around the tobacco field where there is organic fertilizer.

##### Etymology.

The name refers to the type locality, Henan Province in China.

##### Remarks.

*Drawida sinensis* sp. nov. is similar to *D. japonica* s. s. in terms of the grey body coloration, the location of male pores (in 10/11), and the spermathecal atrium in VIII. However, the new species is distinguished from *D. japonica* s. s. by its tightly coiled spermathecal ducts, long sac-like and small atrium, long, thick, and white prostate gland, and long coiled vas deferens. Conversely, *D. japonica* s. s. has elongated coiled spermathecal ducts, relatively large thumb-like atrium, erect club-shaped prostate glands, and coiled and twisted vas deferens. *Drawida sinensis* sp. nov. can be distinguished from *D. henanensis* sp. nov. by its larger body size (length: 41.8–52 mm vs 24–47 mm; width 3.5–4 mm vs 1.8–3 mm), tighter coiling of the spermathecal ducts, and smaller ovisacs (XII–XV vs XII–XX).

*Drawida sinensis* sp. nov. is distinguished from *D. keikiensis* and *D. moriokaensis* by its unique genital markings in VII–XI and the absence of penis, while the latter two species lack these genital markings and the penis pouch is in 10/11. *D. sinensis* sp. nov., *D. koreana*, and *D. siemsseni* exhibit a maximum similarity in width, reaching 4 mm, while the new species is distinguished by its comparatively shorter body length (length 41.8–52 mm). In contrast, the lengths of the latter two species are 63–100 mm and 110 mm, respectively. *Drawida sinensis* sp. nov. is distinguished from *D. calebi* by the presence of a tightly coiled spermathecal duct and the absence of penis, while the spermathecal duct of *D. calebi* is long and coiled, and the penis is short in 10/11. In addition, the new species exhibits the following characteristics: the clitellum in X–XIII, the ovisacs extend from XII–XV, the vas deferens of the testis sacs is long and coiled, and the spermathecal atrium is small in VIII. In contrast, *D. jeholensis* has its clitellum in IX–XIV, the ovisacs extend to XX, the vas deferens of the testis sacs is short and coiled, and the spermathecal atrium is large in 7/8. *Drawida henanensis* sp. nov. also differs from *D. companio* by the presence of two gizzards in XI–XIII and a long, thick, and white prostate, while *D. companio* is characterized by three gizzards in XII–XIV and two small, white prostates.

#### ﻿ Drawida japonica

Taxon classificationAnimaliaMoniligastridaMoniligastridae

(Michaelsen, 1892)

170FB460-DEB5-553F-A81E-7E926CEDF15C

Fig. 4

Moniligaster japonicus Michaelsen, 1892: 232.
?Moniligaster bahamensis Beddard, 1893: 690.
Drawida japonica : Michaelsen 1900: 115.
Drawida japonica typica : Michaelsen 1910: 49.
Drawida japonicus typicus : Michaelsen 1931: 7.
Drawida grahami Gates, 1935: 3; 1939: 408.
Drawida propatula Gates, 1935: 449.
Drawida japonica japonica : Blakemore et al. 2010: 1; Blakemore and Kupriyanova 2010: 8.

##### Materials examined.

• Six clitellates: HNLN-GR-I2_36 (COI accession number: PQ288560), HNLN-GR-I3_01−02 (COI accession number: PQ288577, PQ288578), HNLN-GR-I4_15−16 (COI accession number: PQ288586, PQ288587), HNLN-GR-I4_20 (COI accession number: PQ288590), Luoning County (34.4364°N, 111.6368°E), Luoyang Prefecture, Henan Province, China, 2023-05-18, coll. Huifeng Zhao.

##### Diagnosis.

Length 32–61 mm, diameter 2.0–3.8 mm, segments ≤ 131. Body grey. Prostomium prolobous. Clitellum in X–XIII. Setae lumbricine closely paired. Male pores paired in 10/11. Female pore absents. Spermathecal pores small in 7/8 or inconspicuous. Genital markings unpaired in VII–XII. Spermathecae paired in VII–VIII. Testis sacs paired in IX–X, with white prostate glands in X. Ovary chamber in XI, with ovisacs in XII–XVI or XX. Gizzards white, three in XI–XIII.

##### Description.

**Figure 4.**
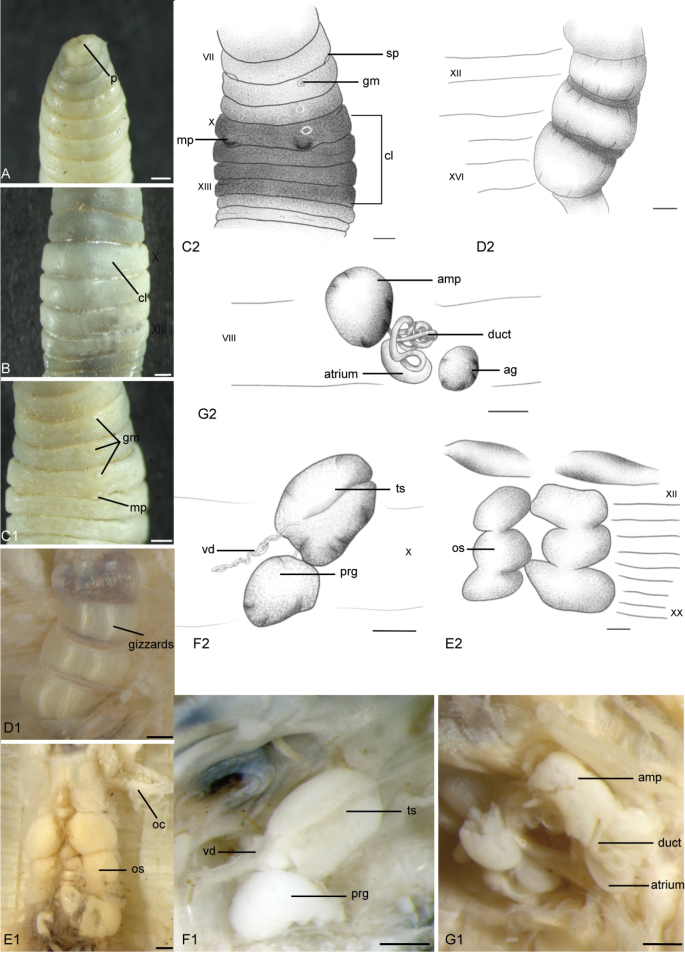
*
Drawida japonica* sensu stricto (Michaelsen, 1892). Luoning, Chinese sample (HNLN-GR-I3_02). **A.** Ventral view of the prostomium; **B.** Dorsal view of clitellum region; **C1, C2.** Ventral view of male pores, genital markings, and spermathecal pores; **D1, D2.** Ventral view of gizzards; **E1, E2.** Ventral view of ovary chamber and ovisacs; **F1, F2.** Right testis sac, vas deferens, and prostate gland; **G1, G2.** Ventral view of spermathecae. Scale bars: 0.5 mm.

***External characters***: Length 32–61 mm. Specimens preserved in alcohol may have varying shades of gray. Clitellum in X–XIII, its color is lighter than the body color (Fig. 4B). Diameter 2–3.8 mm. Segments ≤ 131. Prostomium prolobous (Fig. 4A). Dorsal pores absent. Setae lumbricine, small and closely paired (aa = bc). Male pores protrude in 10/11 between setae b and c, close b (Fig. 4C1, C2). Penis absent. Spermathecal pores are more usually invisible, or small in 7/8 (Fig. 4C2). Female pore absents. Genital markings unpaired and irregular in VII–XII.

***Internal characters***: Septa 5/6–8/9 thick and muscular, 7/8 and 8/9 surround spermathecae, 10/11 and 11/12 relatively thick meet form the ovary chamber. Large yellowish ovisacs extend to XVI or XX (Fig. 4E1, E2). Spermathecae paired in VII–VIII, with small and elliptical ampulla and long coiled ducts, and spermathecal atrium thumb-like and relatively large in VIII (Figs 4G1, G2, 8C). Testis sacs paired in IX–X, with less coiled vas deferens and white prostate glands in X (Fig. 4F1, F2). Penis absent. Three gizzards in XII–XVI, yellowish (Fig. 4D1, D2).

##### Distribution.

Japan (from around Tokyo, Honshu to Nagasaki, Kyushu), China (including Taiwan), Korea (including Quelpart/Jeju-do), India and Pakistan.

##### Remarks.

Morphologically, specimens from China are essentially identical to their Japanese counterparts. However, several minor differences exist between Chinese specimens and those from Japan, such as the body size (32–61 vs 28–70; 2–3.8 vs 2–4.5), the position of the clitellum (X–XIII vs IX, X–XIII, ^1^/_2_XIV), the position of ovisacs (XII–XVI or XX vs XI, XII–XVI), and presence of dorsal pores that absent in Chinese specimens and intermittently present in Japanese specimens. A comprehensive morphological description of the *D. japonica* s. s. specimens from Japan, along with relevant supplementary information is provided in Blakemore and Kupriyanova (2010) and Blakemore et al. (2014).

### ﻿Molecular species delimitation

**Figure 5.**
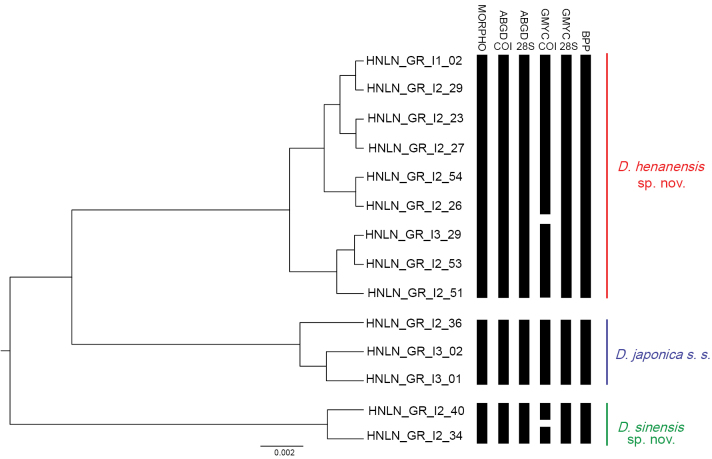
Species delimitation of the *Drawida japonica* species complex. The vertical bar shows the number of species delimited by morphological characteristics and molecular data.

The molecular species delimitation analyses were conducted using three approaches: ASAP, GMYC, and BPP. The results obtained from ASAP and GMYC analyses based on COI and 28S were consistent with the morphological data identifying *D. henanensis* sp. nov., *D. japonica* s. s., and *D. sinensis* sp. nov. as valid species, except for the GMYC analysis based on COI, which oversplit *D. henanensis* sp. nov. and *D. sinensis* sp. nov. (Fig. 5). Furthermore, the BPP analyses of the dataset combining all the gene fragments (COI, 28S, AKAP17 and FLAD1) revealed that three of the delimited species exhibited posterior probabilities of 1.0 or close, aligning with the described morphospecies. In addition, the intra- and inter-specific sequence divergences of the *D. japonica* species complex was calculated using the K2P model based COI (see Table 3). The results indicate that the inter-specific sequence divergence exceeds 15%, and the intra-specific sequence divergence for *D. henanensis* sp. nov., *D. japonica* s. s., and *D. sinensis* sp. nov. ranged from 0–6.5%, 0–9.6%, and 0.5–9.8%, respectively.

### ﻿Phylogenetic relationship of *D. japonica* species complex

**Figure 6.**
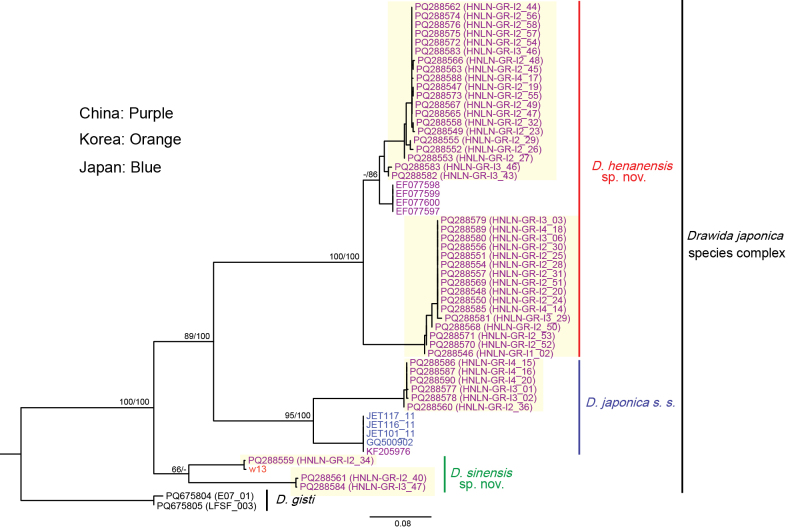
Likelihood tree based on the COI of the *Drawida japonica* species complex using the Maximum Likelihood method and Bayesian Inference. Samples collected in China, South Korea and Japan are highlighted in color; sequences newly generated in this study are indicated by a yellow background. Node labels show maximum-likelihood bootstrap percentages (left slash) and Bayesian posterior probabilities (right slash). Values below 50% for bootstrap and 80% for posterior probability are considered weak support and have been omitted.

**Figure 7.**
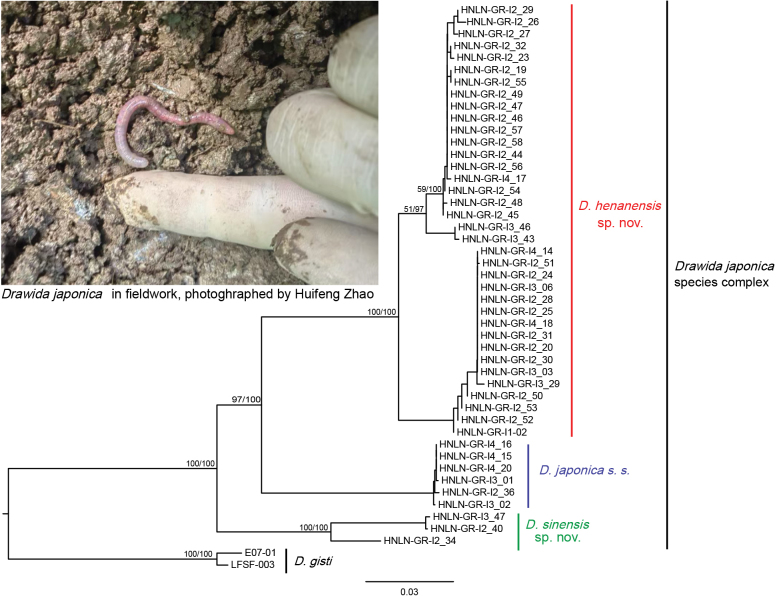
Phylogeny of the *Drawida japonica* species complex reconstructed from the concatenated COI, 28S, AKAP17 and FLAD1 datasets. Node labels show maximum-likelihood bootstrap percentages (left slash) and Bayesian posterior probabilities (right slash). Bootstrap values < 50% and posterior probabilities < 80% are omitted.

The integration of available data from GenBank and relevant literature was employed to reconstruct the phylogenetic relationships of 55 COI sequences of the *D. japonica* species complex, using the ML and BI methods. The phylogenetic tree of COI (Fig. 6) demonstrated that the earthworm samples of this study collected from Luoning (Henan, China) are primarily scattered into three well-supported clades, with a strongly supported bootstrap value (BV) above 85% and a posterior probability (PP) of 100%. The known samples from China (EF077597–EF077600) (Huang et al. 2007), Lake Biwa (JET101, JET116, JET117) (Blakemore et al. 2014) and Hikone (GQ500902) (Blakemore and Kupriyanova 2010) in Japan, as well as the samples from Gyungsanbuk in South Korea (w13) (Blakemore et al. 2014), are scattered in three clades: *D. henanensis* sp. nov., *D. japonica* s. s., and *D. sinensis* sp. nov., respectively (Fig. 6). Furthermore, the results of the phylogenetic analyses provided substantial support, with the BV exceeding 95% and the PP reaching 100% in the combined dataset based on COI, 28S, AKAP17, and FLAD1 (Fig. 7).

**Figure 8.**
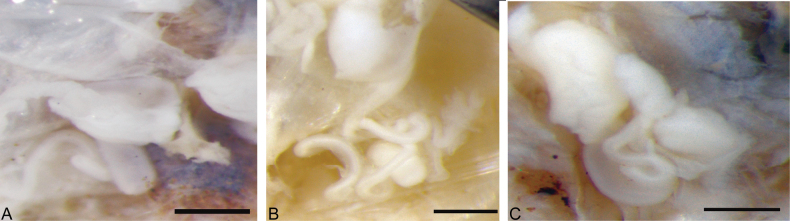
The spermathecal atrium of three species of *Drawida japonica* species complex. **A.***Drawida henanensis* sp. nov. (HNLN-GR-I2_34); **B.***Drawida sinensis* sp. nov. (HNLN-GR-I1_02); **C.***Drawida japonica* (HNLN-GR-I3_02).

## ﻿Discussion

*Drawida* is the most widely distributed genus in the family Moniligastridae, with most of its species confined to their areas of origin in the Indo-Asian region (Narayanan et al. 2024a). Among the various *Drawida* species, *D. japonica* s. l., *D. barwelli*, and *D. nepalensis* are classified as peregrine species (Misirlioğlu et al. 2023; Narayanan et al. 2024a). The distribution of the *D. japonica* species complex has been documented in South and East Asia (Zhang et al. 2012; Kumari et al 2021; Narayanan et al. 2024a; Bi et al. 2025). However, *D. japonica* s. l. possesses one of the weakest locomotive abilities among earthworms, as evidenced by extensive fieldwork conducted by researchers that indicates the possibility of undiscovered species within this species complex.

Traditionally, *D. japonica* s. l. has been considered as a single species, likely due to its extremely similar external morphology and small size, as well as the lack of detailed differentiation in morphological characteristics, leaving its taxonomic status unresolved (Blakemore and Kupriyanova 2010; Blakemore et al. 2014; Kumari et al. 2021; Bora et al. 2024). The use of an integrative taxonomy approach, characterized by the careful examination of both the external and internal morphological characteristics of the species complex in conjunction with the integration of molecular data (including mitochondrial and nuclear markers), facilitates the delineation of more differentiated lineages or species.

Meticulous documentation of external characteristics is imperative. Such examination must include the shape of male pores and spermathecal pores, the presence or absence of penis and female pore, the location of the clitellum, and the number of genital markings. Moreover, certain features, such as setae, male pores, and genital markings, are challenging to discern under a stereomicroscope. For example, in the work of Qin et al. (2025) on the description of new subspecies of *Eisenia nordenskioldi* (Eisen, 1978) from northeastern China, the distance between setae at different body segment positions was explored and showed to be a very useful character for species delimitation. These features may require the use of an electron microscope to obtain clearer images. The following internal characteristics are also of particular importance: the size and shape of the spermathecal ampulla; the length and coiling degree of spermathecal ducts and vas deferens; the shape, location, and size of the atrium; the location of the testis sacs and ovisacs; and the number and location of the gizzards. Notably, certain characteristics, including both external and internal morphological characteristics, are not clearly discernible under a stereomicroscope. Line drawings are employed to elucidate the essential characteristics, including the morphology of the male and spermathecal pores, the degree of coiling of the spermathecal ducts and the vas deferens of testis sacs, and the dimensions and configuration of the spermathecal atrium.

Molecular species delimitation employs DNA data to objectively detect potential independent evolutionary lineages (candidate species) using distance-, monophyly- or coalescent-based models, and then integrates morphological, ecological, and geographical evidence to reach a final taxonomic decision (Padial et al. 2010). This study combined ASAP, GMYC, and BPP for delimitation, revealing that GMYC based solely on COI exhibited excessive splitting (Fig. 5). This discrepancy can be attributed to that COI is a rapidly evolving marker prone to accumulating variation within populations, whereas GMYC relies on branch-length rate conversion, which often misinterprets population differentiation as speciation events rather than genuine species formation (Ballard and Whitlock 2004; Talavera and Vila 2011; Papadopoulou et al. 2013; Zhang et al. 2019). By contrast, 28S exhibits evolutionary conservation and exclusively captures species-level disparities, whereas ASAP utilizes genetic distance barcode gap analysis, a method less susceptible to influence by population-level variation, thereby yielding more robust delimitation outcomes (Carstens et al. 2013; Kapli et al. 2017).

The COI sequences labeled as *D. japonica* which are obtained from China, Japan, and South Korea (Table 1) resolved into three well-supported, reciprocally monophyletic clades—*D. sinensis* sp. nov., *D. japonica* s. s., and *D. henanensis* sp. nov.—demonstrating that the name has been applied to a diversified assemblage rather than to a single species (Fig. 6). Pairwise K2P distances among these clades exceed 15%, a threshold widely regarded as indicative of interspecific divergence within *Drawida* (Huang et al. 2007; Thakur et al. 2021; Nguyen et al. 2022; Liu et al. 2025), and this molecular delimitation is corroborated by discrete morphological characters. Consequently, comprehensive integrative revision of the *D. japonica* species complex is urgently required to delimitate its constituent taxa and clarify their nomenclatural status.

## Supplementary Material

XML Treatment for Drawida henanensis

XML Treatment for Drawida sinensis

XML Treatment for Drawida japonica

## ﻿Additional information

### Use of AI

No use of AI was reported.

### Author ORCIDs

Min Liu https://orcid.org/0009-0002-0090-6219

Zheng Liu https://orcid.org/0000-0002-2631-4302

Nonillon M. Aspe https://orcid.org/0000-0003-0025-6017

Yufeng Zhang https://orcid.org/0000-0002-4066-6355

Huifeng Zhao https://orcid.org/0000-0003-4243-9671

### Data availability

All of the data that support the findings of this study are available in the main text.
